# Enhanced Detection of Cardiac Surgery-Associated Acute Kidney Injury by a Composite Biomarker Panel in Patients with Normal Preoperative Kidney Function

**DOI:** 10.3390/jcdd9070210

**Published:** 2022-07-01

**Authors:** Jurij Matija Kalisnik, Klemen Steblovnik, Eva Hrovat, Ales Jerin, Milan Skitek, Christian Dinges, Theodor Fischlein, Janez Zibert

**Affiliations:** 1 Department of Cardiac Surgery, Paracelsus Medical University, 40791 Nuremberg, Germany; theodor.fischlein@klinikum-nuernberg.de; 2 Medical Faculty, University of Ljubljana, 1000 Ljubljana, Slovenia; 3 Department of Cardiology, University Medical Centre, 1000 Ljubljana, Slovenia; steblovnik@gmail.com; 4 Department of Cardiovascular Surgery, University Medical Centre, 1000 Ljubljana, Slovenia; evahrovat2@gmail.com; 5 Institute of Clinical Chemistry and Biochemistry, University Medical Centre, 1000 Ljubljana, Slovenia; ales.jerin@kclj.si (A.J.); milan.skitek@kclj.si (M.S.); 6 Faculty of Pharmacy, University of Ljubljana, 1000 Ljubljana, Slovenia; 7 Department of Cardiac, Vascular and Endovascular Surgery, Paracelsus Medical University, 5020 Salzburg, Austria; c.dinges@salk.at; 8 Faculty of Health Sciences, University of Ljubljana, 1000 Ljubljana, Slovenia; janez.zibert@zf.uni-lj.si

**Keywords:** acute kidney injury, biomarker, cardiac surgery

## Abstract

We have recently shown that minor subclinical creatinine dynamic changes enable the excellent detection of acute kidney injury (AKI) within 6–12 h after cardiac surgery. The aim of the present study was to examine a combination of neutrophil gelatinase-associated lipocalin (NGAL), cystatin C (CysC) and creatinine for enhanced AKI detection early after cardiac surgery. Elective patients with normal renal function undergoing cardiac surgery using cardiopulmonary bypass were enrolled. Concentrations of plasma NGAL, serum CysC and serum creatinine were determined after the induction of general anesthesia, at the termination of the cardiopulmonary bypass and 2 h thereafter. Out of 119 enrolled patients, 51 (43%) developed AKI. A model utilizing an NGAL, CysC and creatinine triple biomarker panel including sequential relative changes provides a better prediction of cardiac surgery-associated acute kidney injury than any biomarker alone already 2 h after the termination of the cardiopulmonary bypass. The area under the receiver-operator curve was 0.77, sensitivity 77% and specificity 68%.

## 1. Introduction

Cardiac surgery-associated acute kidney injury (CSA-AKI) represents a serious complication increasing morbidity and mortality [1,2,3,4]. Even patients recovering their renal function remain at increased risk of infections in the acute postoperative period and are at higher risk for subsequent (sub)acute and chronic kidney disease progression [5,6].

Contemporary classifications use creatinine concentration and glomerular filtration as criteria for the definition of AKI [7,8]. Due to only the gradual and initially clinically silent rise of serum creatinine levels in the affected patients, delays in diagnosing CSA-AKI up to 48 h after the initial injury are not rare, even when using the most recent Kidney Disease Improving Global Outcomes (KDIGO) [8] criteria. Nevertheless, we have recently shown that assessing subtle creatinine changes as early as in the first 6–12 h after operation, including the biomarker dynamics and not only the absolute values, improves CSA-AKI detection remarkably, especially when combined with artificial intelligence-supported algorithms [9,10].

In hopes of enhancing AKI diagnosis in the window when the injury might still be reversible, novel tubular and functional kidney injury biomarkers have been introduced [11,12,13]. Combinations of various biomarkers and creatinine provided additional information on the severity of clinically expressed AKI as well as an enhanced timely diagnosis [12,14,15].

Thus, the aim of this pilot study was to test the combination of creatinine, NGAL and CysC for the early prediction of CSA-AKI in patients with preoperatively preserved renal function undergoing cardiac surgery.

## 2. Materials and Methods

### 2.1. Study Population

We prospectively enrolled patients undergoing elective aortic valve replacement (AVR) with or without coronary artery bypass grafting (CABG) using cardiopulmonary bypass (CPB) at the Department of Cardiovascular Surgery, University Medical Centre Ljubljana, Slovenia from May 2015 to January 2019. All patients had a normal renal function defined as eGFR > 60 mL/min/1.73 m^2^ and preoperative serum creatinine within normal range. Exclusion criteria were the following: kidney transplant recipients, use of a radiocontrast agent 24 h prior to surgery, emergency cardiac surgery, age less than 18 years, history of previous open-heart surgery and patients who required revision for bleeding or other emergent surgical indication. The Modified Diet in Renal Disease (MDRD) formula was applied for the eGFR calculation: eGFR = 186 × serum creatinine-1.154 × age-0.203 × 1.210 (if black) × 0.742 (if female) [8]. CSA-AKI was defined according to the KDIGO criteria as an increase in creatinine by ≥0.3 mg/dL or by ≥26.5 μmol/L from baseline within 48 h after cardiac surgery or an increase in creatinine to ≥ 1.5 times baseline within 7 days after cardiac surgery [8]. Patient data was collected from the preoperative period until hospital discharge. Patient information, including preoperative characteristics, intraoperative and early postoperative data were retrieved similarly to the protocol of the previous study [13].

### 2.2. Cardiac Procedure Protocol

Selected anesthetic technique depended on the degree of cardiac dysfunction. It consisted of the infusion of intravenous opioids (fentanyl and/or remifentanil) together with an intravenous anesthetic (etomidate as an induction agent and propofol) and in combination with inhalation anesthetics (sevoflurane). A neuromuscular blockade was achieved with a non-depolarizing muscle relaxant (rocuronium). Patients were ventilated to normocapnia. Heparin was administered at a dose of 300 IU/kg (target-activated clotting time ≥ 480 s) and was antagonized with protamine sulfate after the completion of the surgical procedure.

The operations were carried out through median sternotomy in a standardized fashion using CPB (routine ascending aortic cannulation and double stage venous cannula into the right atrium) in mild hypothermia (34–36 °C) and non-pulsatile perfusion mode (50–60 mmHg perfusion pressure and flow rate of 2.4 L/m^2^ per minute). Myocardial protection was achieved by using intermittent anterograde and/or retrograde hyperkalemic cold blood (4 °C) cardioplegia. Patients were transferred to the intensive care unit (ICU) immediately after surgery.

### 2.3. Blood Samples Collection

The determination of creatinine, CysC and NGAL concentrations was performed at the Institute of Clinical Chemistry and Biochemistry, University Medical Centre Ljubljana, Slovenia. Arterial blood was collected at five predetermined timepoints: after the induction of general anesthesia before the surgical incision, at CPB termination and 2 h thereafter, and 24- and 48 h after surgery. Tubes without additives were used for the measurement of creatinine and CysC. For the measurement of NGAL, tubes with ethylenediaminetetraacetic acid were used. Samples were centrifuged to obtain serum or plasma and were then stored at −20 °C for final analyses. The creatinine in the serum was measured using an automated assay based on the modified kinetic Jaffe reaction (Siemens Healthcare Diagnostics Inc., Newark, DE, USA); the limit of detection was 9 µmol/L. For the measurement of CysC in the serum, an ELISA kit (Bio Vendor GmbH, Heidelberg, Germany) with a 0.2 µg/L detection limit was used. The plasma concentration of NGAL was measured with an Advia analyzer (Siemens Healthcare Diagnostics Inc., Newark, DE, USA) using a turbidimetric immunoassay (BioPorto Diagnostics, Gentofte, Denmark); the limit of detection was 12 µg/L.

### 2.4. Statistical Analysis

The data between the AKI groups (Table 1 and Table 2) were compared with the Mann–Whitney U test because the data were non-normally distributed, whereas the differences between the groups for the categorized data were tested with a Chi-square test. The normality of the data was checked with the Shapiro–Wilk test. The data in Table 1 and Table 2 are presented as the median with 1st and 3rd quartile intervals. AKI predictions with the biomarkers analyzed were performed using logistic regression. The dynamics of the biomarker changes over time (deltas) were calculated as Δ1(%) = (End of CPB − preoperative)/preoperative × 100%, Δ2(%) = (2h after CPB − End of CPB)/End of CPB × 100%. Stepwise logistic regression models were created for NGAL, CysC and creatinine preoperatively at the end of CPB and 2 h after CPB with the corresponding deltas. In addition, a logistic regression model was created that included a combination of all three biomarkers. The models were evaluated by a leave-one-out cross validation, whereas the area under the receiver-operating curves (AUCs) with the sensitivity and specificity at optimal thresholds were used as the final evaluation measures. The Transparent Reporting of a multivariable prediction model for Individual Prognosis or Diagnosis (TRIPOD) recommendations were followed under the Type 1b analysis category with the development of the prediction models using the entire dataset with cross validation [16].

All statistical analyses were performed using R version 3.6 (The R Foundation for Statistical Computing, Vienna, Austria; https://www.R-project.org/, accessed on 11 May 2022). Overall, a *p*-value of less than 0.05 was considered statistically significant.

## 3. Results

**Figure 1:**
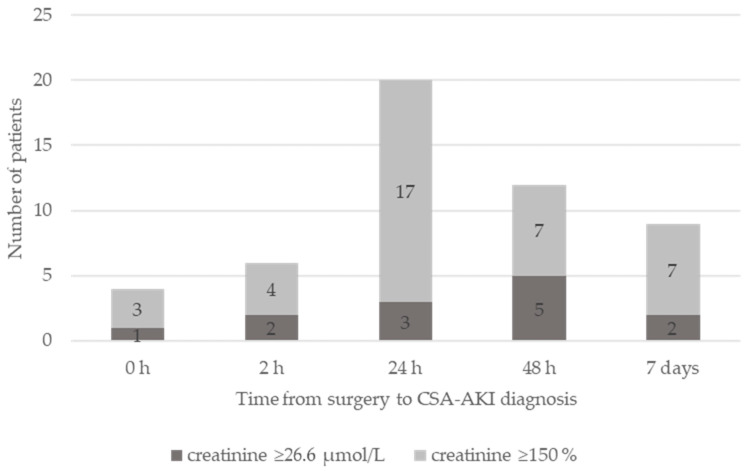
Time from surgery to CSA-AKI diagnosis. The CSA-AKI diagnosis established based on creatinine increase of 26.6 nmol (dark grey) or relative increase of 150% (light grey).

We prospectively analyzed 124 adult patients with normal kidney function who underwent elective cardiac surgery using CPB. Five patients died in the hospital: three were in the AKI group and two were in the non-AKI group (*p* = 0.77). Of the remaining 119, 51 patients (43%) developed AKI. One patient required renal replacement therapy after surgery. Other demographic and preoperative characteristics between the groups are depicted in Table 1. Patients in the AKI group had longer CPB and cross-clamp times, received more red blood cell transfusions, received more fresh frozen plasma units and stayed longer in the ICU (Table 1). From 51 patients, 4 received their diagnosis at the termination of CPB, 6 patients 2 h thereafter, 20 patients 24 h after surgery, 12 at 48 h after surgery and 9 patients within 7 days of surgery. The CSA-AKI diagnosis was established using the criterium of a creatinine increase of 26.6 nmol in 13 cases and in the remaining 38 using the criterion of a relative increase of 150% as depicted in Figure 1.

As depicted in Table 2, the NGAL concentrations reached their maximum at CPB termination in both the AKI and non-AKI groups. While not distinguishing between the groups preoperatively, the NGAL concentration was higher in the AKI group at CPB termination and two hours thereafter. No statistically significant differences in the relative NGAL concentration changes over time were observed (Table 2).

Similar to NGAL, the CysC concentration was higher in the AKI group at CPB termination as well as at the arrival to the ICU. The relative CysC changes remained similar across the groups at all timepoints (Table 2).

Although the absolute creatinine concentrations did not differ between the AKI and non-AKI group at any time, we observed a highly significant relative increase in creatinine at CPB termination with respect to the preoperative level in the AKI group (Table 2). Of note, the relative creatinine increase was significant, although the absolute creatinine concentrations were similar and considered within normal range.

**Figure 2:**
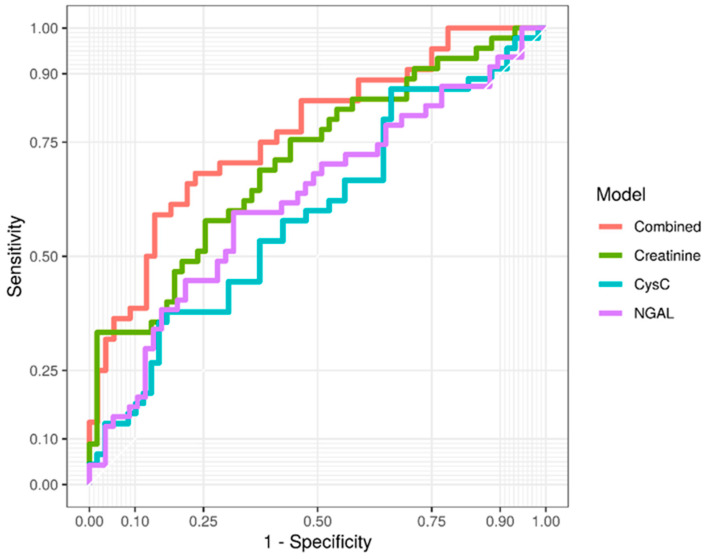
ROC curves for creatinine, cystatin C, NGAL and combined model.

As depicted in Table 3, single biomarker models for predicting CSA-AKI using concentrations and their relative changes over time for NGAL, CysC and creatinine performed worse than the combined model of all three biomarkers. According to receiver operating characteristics, a creatinine-based model offered the best discriminatory power among “stand-alone” models (AUC 71%) for CSA-AKI while NGAL (AUC 63%) and CysC (AUC 59%) exhibited a poor discriminatory capability (Table 3). On the contrary, a combined model utilizing all three biomarkers along with their sequential relative changes performed with the highest discriminatory power (AUC 77%) for CSA-AKI as early as two hours after CPB termination, with a sensitivity of 77% and a specificity of 68% (Table 3, Figure 2).

## 4. Discussion

Our prospective study demonstrated that the biomarkers NGAL and CysC enhance early CSA-AKI detection after cardiac surgery among patients with normal preoperative kidney function. Moreover, by determining NGAL and CysC in conjunction with creatinine, the identification of patients prone to CSA-AKI was made possible within the first two hours after surgery. These preliminary results are in harmony with our assertions that anticipated the further improvement of CSA-AKI by combining NGAL, CysC and creatinine serum concentrations as well as their changes over time [17].

It is possible to reduce the frequency and severity of CSA-AKI [18] with the implementation of the KDIGO guidelines or with a timely application of novel therapeutic approaches, especially in properly identified high-risk CSA-AKI patients [18,19,20]. Seemingly, the broadest spectrum of cardiac surgery patients can be effectively screened to detect CSA-AKI in the early postoperative hours to enable effective interventions within the early therapeutic window for the optimally selected patients (10, 18). We demonstrate a promising utilization of a triple biomarker panel to further narrow the window between the end of surgery and the timepoint of a reliable CSA-AKI diagnosis, from 6–12 h (10) to possibly less than one hour after surgery. The latter might be achieved with a relatively simple setup utilizing routine blood postoperative sampling and on-line diagnostics with the selection of biomarkers that are available at an appealing price-to-performance ratio.

As noted in the model using the determination of creatinine and an early marker of the tubular damage biomarker neutrophil gelatinase-associated lipocalin (NGAL) and Cystatin C (CysC), we underline that including the dynamics of early changes and not only the absolute values remarkably improved early CSA-AKI detection.

### 4.1. Neutrophil Gelatinase-Associated Lipocalin

Our results are in concordance with previous studies that established NGAL, a kidney cellular damage biomarker, as an early marker for CSA-AKI [21,22]. Contrary to Troponin for myocardial damage, NGAL has not yet been fully implemented into early renal damage diagnostic practice due to vastly diverging cut-off values from 150 µg/L [23] to 290 µg/L [24]. A recent meta-analysis showed that the different population settings were the main source of heterogeneity [11]. It has also been suggested that NGAL may be more discriminant in de novo AKI rather than in AKI superimposed on chronic kidney disease [12]. Moreover, sequential NGAL measurements have been proposed to further enhance the diagnostics [25]. Nonetheless, only NGAL dynamics from preoperative samples to one day after surgery have been mostly studied [25]. Unlike the others, we focused on the first postoperative hours in our study in hope of allowing for an earlier diagnosis, potentially maximizing the effects of preventative measures for CSA-AKI reduction.

### 4.2. Cystatin C

Similar to creatinine, CysC is a biomarker of kidney function. Like NGAL, CysC is not widely used in clinical practice. Current studies remain unclear about the benefit of CysC for the early prediction of CSA-AKI. Early studies reporting the performance of plasma CysC showed good correlations with AKI in the AUC range of 0.8–0.9 [26,27]. These findings were challenged by several studies reporting that the performance of CysC for AKI prediction was poor, in the range of 0.6–0.7, remarkably disputing the anticipated utility of CysC, especially if the definition of AKI was creatinine-based [26]. However, CysC performed better at predicting more severe AKI (RIFLE ≥ R) [18]. While the mean reference serum cystatin C level is 840 µg/L in non-Hispanic adolescents with normal renal function [26], the reported cut-offs for the development of AKI stage 1 or higher and RIFLE stage R or higher were 1100 µg/L (AUC 0.76, sensitivity 74% and specificity 67%) and 1240 µg/L (AUC 0.69, sensitivity 76% and specificity 63%), respectively [26,28]. Early postoperative (10 h after surgery) CysC has previously been associated with CSA-AKI [29]. Although in the reported normal range, we found CysC to be significantly higher at the CPB termination as well as 2 h after the end of CPB in patients developing CSA-AKI.

### 4.3. Creatinine

A longer duration and an advanced stage of CSA-AKI decreases the likelihood of renal recovery and increases the risk for end-stage renal disease [14,30]. It is therefore important to detect patients at greater risk of developing AKI as early as possible. Due to its dynamic, absolute concentration of creatinine after surgery is an absolute late discriminator compared to CysC and NGAL and does not offer a good diagnostic value on arrival to the ICU [23,30,31]. However, new data shows that even small increments of creatinine early after surgery can predict the development of AKI [9,10,28,32]. We speculated that, combined with NGAL and CysC, a triple biomarker model including creatinine could provide a simple fine-tuning tool for the prediction of CSA-AKI in the very early preclinical stage [17]. Indeed, the present study confirms our previous observations that even small incremental creatinine changes in the absence of a clinically relevant absolute creatinine increase provide a solid, often overseen basis for early CSA-AKI diagnosis [9,10].

### 4.4. Combined Model

Several previous studies have shown that combining creatinine and other functional or kidney damage biomarkers could improve the prediction of CSA-AKI compared to single biomarkers [28,30,33]. The present study utilizes a combination of NGAL, CysC and creatinine values and their relative changes over time early in the postoperative course offering a fair-to-good predictive value e for CSA-AKI at an early stage after surgery. The latter upgrades our creatinine-based models [9,10] for early CSA-AKI prediction and possibly unveils the yet unused potential of composite biomarker panels for the early diagnosis and prevention of CSA-AKI, preferably by reducing “detection time” from 12 hours after surgery to only a few hours after surgery.

### 4.5. Strengths and Limitations

As this is a single-center study, conclusions regarding clinical risk classification for AKI are limited. The predictive ability of biomarkers was comparable to other similar studies in the field [15,23,34]. However, this study was deliberately restricted to patients after aortic valve replacement with or without coronary artery bypass surgery with normal preoperative kidney function and may not be generalizable to the general cardiac surgery population. Because of the relatively small sample size, we evaluated our models with a leave-one-out cross validation to obtain more unbiased results, but on the other hand, we cannot calculate confidence intervals for the AUC values. Further, the study primarily aimed at the determination of biomarkers in blood, so urinary proteins as a potential marker of kidney damage were not assessed neither pre- nor postoperatively. On the other hand, our study has several strengths. This study focused on the early concomitant determination of the tubular injury marker NGAL as well as the glomerular filtration rate marker CysC for CSA-AKI with parallel conventional creatinine measurements during the preoperative and early postoperative period when specific and timely interventions may result in a significant clinical and financial benefit. Furthermore, serum or plasma renal markers may be of value as they are independent of the availability and the amount of urine production in the early intra- and postoperative period.

## 5. Conclusions

A prediction model using NGAL, CysC and creatinine concentrations and their relative changes over time provides the best-so-far reported CSA-AKI prediction already two hours after CPB termination with an AUC of 0.77, sensitivity of 77% and specificity of 68%. The conclusions reached need to be validated in a larger prospective trial.

## Figures and Tables

**Table 1:** Patient Characteristics.

	Non-AKI	AKI	*p*-Value
	*n* = 68	*n* = 51	
Demographic and preoperative characteristics			
Age (years)	72.5 [62.9;79.0]	75.4 [67.5;81.3]	0.080
Male gender, n (%)	44 (64.7%)	31 (60.8%)	0.805
BMI (kg/m2)	27.0 [23.9;30.0]	27.8 [24.7;30.3]	0.309
Diabetes mellitus (non/oral/insulin)	53/11/4	31/13/7	0.109
Arterial hypertension, n (%)	53 (77.9%)	44 (86.3%)	0.357
Hypercholesterolemia, n (%)	44 (64.7%)	29 (56.9%)	0.497
Left ventricular EF (%)	60.0 [55.0;65.0]	60.0 [55.0;61.5]	0.238
ACEI or ARB	41(60.3%)	31(60.8%)	1.000
Preoperative creatinine (µmol/L)	80.0 [67.8;90.0]	69.5 [54.8;90.8]	0.085
Preoperative eGFR [mL/min/1.73 m^2^]	101 [74.6;115]	84.6 [68.5;99.9]	0.193
Intraoperative and postoperative characteristics			
Combined surgery (AVR + CABG), n (%)	11 (16.2%)	16 (31.4%)	0.102
CPB time (min)	84.0 [65.0;113]	100 [71.5;124]	0.061
Cross-clamp time (min)	62.0 [45.2;83.5]	75.0 [55.0;97.0]	0.029
Units of RBC transfusion	0.00 [0.00;2.00]	2.00 [1.00;4.00]	<0.001
Units of FFP transfusion	0.00 [0.00;2.00]	0.00 [0.00;3.00]	0.104
Platelets transfusion, 0/1/2 (%)	64/4/0 (94/6/0%)	45/4/1 (90/8/2%)	0.572
Respiratory support (h)	12.0 [7.75;16.0]	14.0 [8.00;20.5]	0.206
Inotropes (h)	0.00 [0.00;21.5]	3.00 [0.00;46.5]	0.247
ICU stay (days)	3.00 [1.00;4.00]	4.00 [1.00;8.50]	0.018
Hospital stay (days)	8.17 [6.25;12.1]	9.19 [7.00;20.0]	0.106

Data are presented as median [Q1, Q3]. ACEI = Angiotensin-converting enzyme inhibitors; AKI = acute kidney injury; ARB = Angiotensin II receptor blockers; BMI = body mass index; AVR = aortic valve replacement; CABG = coronary artery bypass grafting; CPB = cardiopulmonary bypass; EF = ejection fraction; eGFR = estimated glomerular filtration rate; ICU = intensive care unit; RBC = red blood cell; and FFP = fresh frozen plasma. Statistically significant difference between the groups is considered at *p* < 0.05.

**Table 2:** Serum/plasma creatinine, CysC and NGAL values in non-AKI and AKI groups.

		Non-AKI	AKI	*p*-Value
		*n* = 68	*n* = 51	
NGAL (µg/L)	Preoperative	60.0 [36;105]	87.0 [59;117]	0.085
	End of CPB	144 [114;190]	196 [156;260]	0.002
	2 h after CPB	122 [88;186]	177 [154;224]	<0.001
Δ [%]	Δ1 (End of CPB—preoperative)	140 [64;249]	134 [68;229]	0.899
	Δ2 (2 h after CPB—End of CPB)	−14.55 [−30;3]	−4.09 [−18;13]	0.065
CysC (µg/L)	Preoperative	795 [683;922]	857 [716;1175]	0.125
	End of CPB	740 [632;886]	851 [723;998]	0.024
	2 h after CPB	748 [649;914]	857 [677;1086]	0.031
Δ [%]	Δ1 (End of CPB—Preoperative)	−0.53 [−20;15]	3.18 [−14;23]	0.317
	Δ2 (2 h post CPB—End of CPB)	−1.36 [−19;13]	−2.36 [−16;16]	0.740
Creatinine (µmol/L)	Preoperative	80.0 [68;90]	69.5 [55;91]	0.085
	End of CPB	71.0 [60;87]	81.5 [65;96]	0.128
	2 h after CPB	79.0 [69;92]	87.0 [70;100]	0.291
Δ [%]	Δ1 (End of CPB -preoperative)	−3.37 [−18;7]	8.53 [−7;28]	<0.001
	Δ2 (2 h post CPB- End of CPB)	11.5 [3;21]	7.94 [0;19]	0.512

Data are represented as median [Q1, Q3]. Statistically significant difference between the groups is considered at *p* < 0.05. AKI = acute kidney injury; CPB = cardiopulmonary bypass; CysC = cystatin C; and NGAL = neutrophil gelatinase-associated lipocalin.

**Table 3:** Modeling of CSA-AKI prediction.

NGAL Model	CysC Model	Creatinine Model	Combined Model
PreopNGAL, NGAL end of CPB *, NGAL 2 h after CPB, Δ1 and Δ2	PreopCysC *, CysC end of CPB, CysC 2 h after CPB, Δ1 * and Δ2	PreopCREAT, CREAT end of CPB *, CREAT 2 h after CPB, Δ1 * and Δ2	CysC end of CPB *, CysC Δ1 *, NGAL 2 h after CPB *, CREAT Δ1 *
AUC (%): 63	AUC (%): 59	AUC (%): 71	AUC (%): 77
Sensitivity: 74%	Sensitivity: 78%	Sensitivity: 76%	Sensitivity: 77%
Specificity: 45%	Specificity: 38%	Specificity: 51%	Specificity: 68%

* Statistically significant variables in the logistic regression model. Note that in the combined model only significant variables are reported. AUC = area under the curve, sensitivity and specificity values are reported at optimal cut-off points; CPB = cardiopulmonary bypass; CysC = cystatin C; NGAL = neutrophil gelatinase-associated lipocalin; and PreOP = preoperatively.

## Data Availability

The data presented in this study are available on request from the corresponding author.
